# Exposure to Environmental Contaminants and Lung Function in Adolescents—Is There a Link?

**DOI:** 10.3390/ijerph15071352

**Published:** 2018-06-27

**Authors:** Marike M. Leijs, Janna G. Koppe, Kees Olie, Pim de Voogt, Wim M. C. van Aalderen, Gavin W. ten Tusscher

**Affiliations:** 1 Department of Paediatrics and Neonatology, Emma Children’s Hospital Amsterdam University Medical Center, 1105 AZ Amsterdam, The Netherlands; janna.koppe@inter.nl.net (J.G.K.); w.m.vanaalderen@amc.uva.nl (W.M.C.v.A.); 2 Ecobaby Foundation, Hollandstraat 6, 3634 AT Loenersloot, The Netherlands; 3 Department of Dermatology and Allergology, RTWH University Aachen, Pauwelstrasse 30, 52074 Aachen, Germany; 4 IBED/ESPM, University of Amsterdam, 1090 GE Amsterdam, The Netherlands; k.olie@upcmail.nl (K.O.); w.p.devoogt@uva.nl (P.d.V.); 5 KWR Watercycle Research Institute, P.O. Box 1072, 3430 BB Nieuwegein, The Netherlands; 6 Department of Paediatrics and Neonatology, Westfriesgasthuis, Maelsonstraat 3, 1624 NP Hoorn, The Netherlands; g.w.ten.tusscher@planet.nl

**Keywords:** dioxin, PCB, BDE, endocrine disruptors, childhood, prenatal exposure, longitudinal study, lung function

## Abstract

Dioxins (polychlorinated dibenzo-p-dioxins (PCDDs) and polychlorinated dibenzofurans (PCDF)), polychlorinated biphenyls (PCBs), and brominated flame retardants (BDEs) are well known toxic environmental contaminants. Their possible role in the incidence of respiratory disease is not yet well understood. Previous studies showed a negative effect on lung function in relation to prenatal and lactational dioxin exposure in pre-pubertal children. Effects of BDE exposure on the lung function have not previously been evaluated. As part of a longitudinal cohort study, the effects of perinatal dioxin (PCDD/F) exposure and serum PCDD/F, dl-PCB, and BDE levels on lung function in adolescents were assessed using spirometry, a body box, and diffusion measurements. Thirty-three children (born between 1986 and 1991) consented to the current follow-up study. Prenatal, lactational, and current dioxin, PCB, and BDE concentrations were determined using GC-MS. No relationship was seen between prenatal and lactational dioxin exposure, nor with current PCB body burden, and lung function. Indications of increasing airway obstruction were seen in relation to increasing current BDE exposure. This is a novel finding and certainly warrants further research.

## 1. Introduction

The increasing incidence of respiratory disease throughout the (Western) world is not yet fully understood. While genetic factors certainly play a role, they are not a sufficient explanation [1,2]. Over the last decades it has been commonly accepted that environmental pollutants play a role in respiratory disease [1,3]. The association between asbestos exposure and mesothelioma, as well as between smoking or exposure to fine particles and chronic obstructive pulmonary disease (COPD) is no longer controversial [3,4]. Environmental factors, such as air pollution and food contamination, have received more attention the last years. In this manuscript we consider some widespread specific environmental contaminants in relation to lung function.

Polychlorinated dibenzo-p-dioxins (PCDDs) and polychlorinated dibenzofurans (PCDFs) (henceforth jointly referred to as dioxins) belong to the group of the most toxic manmade substances known, and have been associated with malignancy, congenital malformations, immunosuppression, and respiratory disorders [5,6,7,8]. Dioxins are formed among others as by-products of waste combustion processes, such as municipal incinerators. They are poorly degradable in nature, persist in the environment, enter our food chain via fish-oils and animal fats, and are primarily stored in adipose tissues, the result of their lipophilicity [9,10]. This lipophilicity allows them to readily pass into a mother’s breast milk and to readily pass the placenta, whereupon they are stored in fetal adipose tissues [11,12,13,14]. Thus, unborn children and breastfed children are exposed to relatively high “background” dioxin levels.

Another group of compounds, brominated diphenylethers (BDEs), have been widely used over the last few decades as flame retardants in various materials such as electronic equipment, plastics, carpet liners, and textiles. Humans are exposed to BDEs mainly by ingestion and inhalation. Numerous recent publications have shown the presence of BDEs in dust, also in homes [15]. Since the early 1990s, BDEs have been measured in the serum of humans. Currently, levels of these compounds are frequently encountered in humans all over the world [16,17].

In an earlier follow-up of our cohort, a negative association was found between prenatal and lactational dioxin exposure and the FEV1/FVC ratio, an indicator for lung obstruction [18], showing that perinatal exposure to dioxins and related compounds may have consequences spanning many years. Moreover, in both animals and humans, the respiratory system is a target of dioxin-toxicity [18,19].

In our longitudinal cohort study of the development of children with known perinatal dioxin exposure, we therefore again assessed the lung function of the study participants during the pubertal period using spirometry and a detailed medical history.

## 2. Materials and Methods

### 2.1. Study Population

This study is part of a longitudinal cohort study of 14–19 year old children, studied during their neonatal (*n* = 60) [20], toddler (*n* = 60) [21], and pre-pubertal period (*n* = 41) [18,22]. All of the 33 children (18 girls and 15 boys) who participated in the current follow-up were born in the Amsterdam/Zaandam region of the Netherlands. Twenty-five of the children were still inhabitants of the region at the time of the current follow-up. All subjects were Caucasian. Dioxin exposure was previously determined in the perinatal period in breast milk of the mothers. From the total cohort of 41 subjects who participated in the pre-pubertal study, one subject suffered from an Ewing sarcoma and was excluded from the current follow-up. One subject was partly excluded because of an extra Y chromosome. Five subjects declined to participate in the new follow-up, three could not be traced.

After exclusion of the above-mentioned subjects, 33 adolescents underwent a lung function test.

Of these 33 subjects, 2 refused to undergo vena puncture and 2 refused a second vena puncture after blood clotting in the first needle.

The study was performed according to the principles of Good Clinical Practice and approved by the institutional ethics review board (05/003#05.17.0438). All participants of the study and their parents signed an informed consent form.

### 2.2. Medical Examination

A medical history was taken by one and the same physician (MML) using a questionnaire which included a history of asthma and smoking habits. The questionnaire also included general questions regarding the pubertal development and other reproductive features and general information such as school and electives/vocation (e.g., agricultural with possible pesticide exposure) and to assess unusual food intake.

### 2.3. Lung Function

Lung function parameters that were obtained and evaluated were spirometric values ((VC MAX (maximum vital capacity), FVC (forced vital capacity), FEV1 forced expiratory volume in 1 s, FEV1/VC MAX, PEF: (peak expiratory flow), FEF50: (forced expiratory flow 50%)), , diffusion measurements (TLCO SB (transfer factor for carbon monoxide), VA (alveolar volume), VIN (inspired volume)), and measurements in the body box (VC MAX, TLC (total lung capacity), RV (rest volume), RV%TLC, FRC (functional residual capacity)). European Respiratory Society (ERS) principles were adhered to (ERS, 1993).

Lung function measurements were performed using the Masterscreen PFT plus bodybox (Jaeger Viasys, Germany). A standardized protocol was used and at least 3 technically correct maneuvers were performed. Short- and long-acting beta2-agonists were stopped 12 h before lung function measurements. The lung function outcomes were compared with reference values developed by Zapletal et al. [23]. Normal values were corrected for height and weight. The validity of all the lung function tests was evaluated by a pediatric pulmonologist (WvA) blinded to the other outcomes.

### 2.4. Laboratory Analyses

For measurements of the PCDD/F, dl-PCB, and BDE serum concentrations, 30 subjects underwent vena puncture, following a fasting period of at least four hours. The serum was isolated from the blood samples and the samples stored at −20 °C until analysis. Perinatal PCDD/F levels and serum levels of PCDD/Fs, PCBs, and BDEs were determined in an uncontaminated laboratory at the Institute for Biodiversity and Ecosystem Dynamics of the University of Amsterdam. We measured the serum levels of the 17 most toxic dioxin congeners (seven PCDDs and ten PCDFs) (see Table 1). In addition, the concentration of 3 dioxin-like PCBs (77, 126, 169) and 8.

BDEs (28, 47, 99, 100, 153, 154 and183) were measured. The concentrations of dioxin and dioxin-like PCB (dl-PCB) congeners were expressed in toxic equivalents (TEQ) ng/kg fat. The prenatal dioxin exposure was previously measured in the mother’s milk 3–4 weeks after birth, representing the dioxin exposure before birth. The postnatal/lactational exposure represents the prenatal exposure multiplied by the total breast milk intake whereby the cumulative total lactational exposure was determined [24].

The extracted lipids from the blood serum were determined gravimetrically and calculated using the formula: Lipids: 0.92 + 1.31 (cholesterol + triglycerides) [25]. An activated carbon column (Carbosphere) was used as the first clean-up step. The dioxin fraction was isolated, and a further clean-up was performed using a column of AgNO_3_ on silica gel and a column of activated Al2O3 on silica gel. Polybrominated diphenylether fractions were purified using acid-base silica columns and activated aluminum oxide. Detection and quantification were performed after concentrating the sample and using GC-high resolution MS [17]. As an internal standard, we used a mixture of 13C-labelled PCDD/Fs, PCBs, and BDEs.

### 2.5. Statistical Analyses

For statistical analyses we used the software package SPSS^®^. We used the multiple linear regression for the correlations between the lung function parameter and the PCDD/F- or dl-PCB- or totalTEQ-(PDCC/Fs and dl-PCB) levels. For calculating the normality of the distribution of the dependent variables, the Kolmogorov–Smirnov test was used. A normal distribution was found for all of the outcome variables (the lung function parameters).

If the scatterplot between the independent and outcome variables showed neither a linear nor curvilinear relationship, we used Spearman’s correlation coefficient (ρ).

As dependent (or outcome) values we used spirometric values (VC MAX, FEV 1, FEV1/VC MAX, PEF, FEF 50), diffusion measurements (TLCO SB, VA, VIN), and measurements in the body box (VC MAX, TLC, RV, RV%TLC, FRC). The levels of serum dioxins (PCDD/Fs), dl-PCBs, and serum BDEs were the independent variables.

We used age as a co-factor in the multiple linear regression models. We calculated the standardized regression coefficient to calculate the relative strength of the independent variable. The level of significance was 5% (p = 0.05).

## 3. Results

Table 1 shows the descriptive statistics of the cohort. Serum PCDD/F levels were not significantly correlated to serum dl-PCB or BDE levels, neither to prenatal or lactational levels.

### 3.1. Prenatal and Lactational Dioxin Exposure in Relation to Lung Function

No negative relationship was seen between FEV1/FVC and prenatal nor with lactational PCDD/F exposure. We found no significant associations with the other lung function parameters either (data not shown).

### 3.2. Serum Dioxin, dl-PCBs, and Total TEQ in Relation to Lung Function

Surprisingly, we found a positive correlation between the current serum dioxin levels as well as the current total TEQ levels and some of the measured lung function parameters (VC MAX, FEV1 and VIN), and one borderline negative relationship with RV%TLC (β: −0.45, p: 0.052). Gender was a co-factor in this statistical model. See Table 2.

For the dl-PCBs no correlation was found with the lung function parameters. Other (non-dioxin-like) PCBs were not assessed in this study.

### 3.3. Serum BDEs in Relation to Lung Function

**Figure 1:**
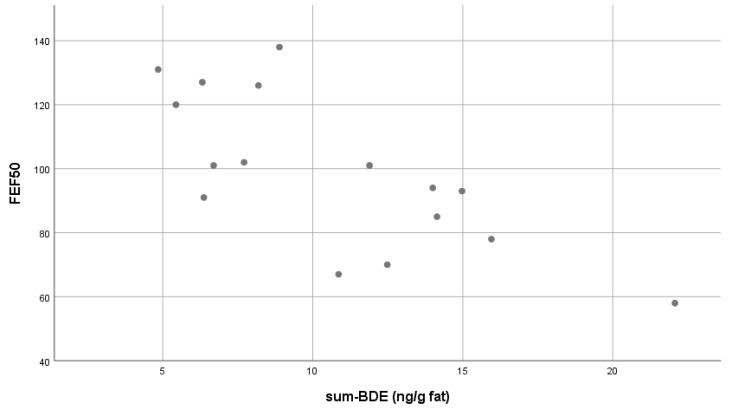
FEF50 in correlation with the sum-BDE levels ^a^. (^a^ In the graphic the outlier with very high BDE levels was excluded).

We found a significant correlation between the sum BDE and some of the lung function parameters. For the spirometry test, FEV1 was significantly correlated ((ρ): −0.539, p: 0.032), as well as FEV1VCMAX ((ρ): −0.575, p: 0.02), and FEF 50 ((ρ): −0.699, p: 0.003) (see Table 3 and Figure 1). For the diffusion parameters or body box measurements, no correlation was found. Excluding one outlier with a very high sumBDE level, did not influence the significance of the correlations. Gender had no influence on the significance of the outcomes.

## 4. Discussion

This follow-up study suggests that brominated diphenyl ethers may negatively affect lung function in teenagers. In the current study, we found no decrease in lung function in correlation to prenatal or lactational dioxin exposure, as seen in a previous study during earlier childhood.

While it has long been known that environmental factors influence lung function, available data are generally still limited. Much has been documented about tobacco smoke, asbestos exposure, and air pollution, but data on other environmental chemicals is much scarcer. Occupational medicine, while being important, cannot freely be extrapolated for background environmental exposure. In occupational medicine far higher exposures are generally seen than in background exposures. Furthermore, the former exposure is generally acute whilst background exposure is generally chronic. Finally, exposure in children occurs during critical developmental windows, possibly resulting in other effects than in adults [5,26].

### 4.1. Accidents

Various major accidents, resulting in large populations being exposed to high concentrations of dioxins, PCBs, and furans have occurred. Polychlorinated biphenyl, dioxin, and furan exposure following rice oil contamination in Japan in 1968 caused chronic bronchitis in 40% of the exposed and persistent suboptimal lung function [27]. A similar incident in 1979 on the island of Taiwan led to 25% of the highly exposed babies dying within four years after birth as a result of respiratory disorders. Respiratory distress and pneumonia during the first six months of life were common [28]. An explosion at an Italian chemical plant in Seveso in 1976 led to high dioxin exposure, causing an increased mortality risk for respiratory disease, mainly chronic obstructive pulmonary diseases (COPD) [29]. However, it must be borne in mind that, contrary to our study, these accidents were the result of an acute toxic effect and not of a developmental effect.

### 4.2. Dioxins and PCBs

In contrast to the prepubertal study, no relationship between perinatal dioxin exposure and lung function was seen during the puberty follow-up. This could possibly point to an improvement in the lung function deficit following further clearance of the relatively high perinatal exposure. The half-life of dioxins is estimated at 7–12 years, but may be shorter [30]. The cohort was aged 14–19 years at the current follow-up.

Another study showed that the prenatal and lactational dioxin and PCB exposures are around 26 times higher than later ones, when the intake of animal fats and human breast milk is far lower [31]. This was also seen in our study, where the measured serum dioxin and PCB levels were much lower than during the prenatal and lactational exposure [17].

For the correlation of lung function parameters with environmental pollutants only a few studies can be found. As mentioned above in a previous study in our cohort (mean age of the cohort 8.2 y), a correlation between airway obstruction (FEV1/FVC) and prenatal and lactational PCDD/F exposure was seen [18].

Another study found that maternal concentrations of persistent organic pollutants (POPs) (PCBs, hexachlorobenzene (HCB), and dichlorodiphenyldichloroethylene (p,p’-DDE)were positively related to offspring airway obstruction (FEV1/FVC < 75%). No associations were observed with reduced lung function (FEV1 % of predicted value < 90%). No correlations with allergic sensitization were found [32].

In contrast, a German study found no significant differences in static and dynamic lung function parameters between investigated areas with slightly differing DDE, PCBs, and PCDD/PCDF levels in 10-year-old children [33].

In another longitudinal study, prenatal DDE was associated with wheeze at age four years but not thereafter. Prenatal hexachlorobenzene (HCB, another dioxin-like environmental pollutant) was associated with wheeze and chest infections at age 10 years. No associations were found with sum PCB levels [34]

In early childhood (0–3 years) prenatal dietary exposure to PCBs and dioxins may increase the risk of wheeze and the susceptibility to infectious diseases in early childhood [35].

In our group, however, the children were examined in another age category, where other co-factors (other environmental pollutants and also small particle air pollution) might play a bigger role, and the level of exposure to dioxins was decreasing.

How environmental contaminants like PCBs, BDEs, and dioxins might interfere with the lung function remains unclear [36]. As a possible role in reduced lung function, a dysregulation of immunological markers like IL-10 is suggested [34]. Another study showed that IL-24 is a pulmonary exposure target cytokine of the environmental AhR agonist benzo(a)pyrene [37].

The limited subjects in the current follow-up may also lead to wrong conclusions. However, the current study shows no trend with the lung function parameters in the scatter diagrams, in relation to the prenatal or lactational PCDD/F exposure; one would expect a trend, even if not significant, with the number of subjects taking part.

Finally, it is possible that spirometry and body box measurements are not sensitive enough to detect small deficits still present, following perinatal exposure.

At this stage we must conclude that the previous lung function deficit, related to prenatal and lactational dioxin exposure, is no longer seen. We hypothesize that environmental factors (exposure to other endocrine disruptors, small particles pollution) other than dioxins now play a bigger role.

As mentioned above, a weakness of the study is the limited number of studied subjects. In addition, besides the measured PCDD/Fs, PCBs, and BDEs, other endocrine disruptors not assessed, as well as small particle pollution, could also affect lung function outcomes.

### 4.3. Brominated Diphenyl Ethers

To our knowledge we are the first to link serum BDE levels to lung function deficits. We measured BDE levels in the serum of the adolescents. The perinatal levels are not known.

It is alarming to find a relationship between serum BDEs and lung functioning. Brominated diphenyl ethers are very commonly used substances worldwide. Until about 2005, human serum levels were increasing [38,39]. Currently, levels are decreasing, however, levels of Firemaster 550 components (EH-TBB, BEH-TEBP, and TPHP) in house dust were reported to be higher in 2011 than in 2006, and this flame retardant is suggested as a replacement for BDEs [40].

Furthermore, indoor dust contains far higher concentrations of BDEs than outdoor air [41]. Other studies have linked BDE exposure with endocrine disruption and neurotoxicity [42,43,44,45,46]. However, we found no study which evaluated effects of these compounds on lung function in vivo or lung cells in vivo or in vitro. Further scientific evidence of adverse health effects is still wanting or unexplored.

The lung function outcomes would seem to plead for an increased obstructive component in the respiratory system, as a result of the BDE exposure. An increase in an obstructive component would suggest an increase in asthmatic complaints. It is then interesting to note that the enormous increase in the incidence of asthma during the 80s and 90s of the previous century, were at a time when the use of (and exposure to) BDEs rapidly increased worldwide, especially in the Western world. This prompts us to hypothesize that at least a portion of the increased incidence of asthma throughout the Western world may be the result of increasing BDE exposure. However, the size of our current cohort limits us in evaluating asthma and BDE separately.

In addition, it must be borne in mind that obstructive lung disease encompasses more than asthma. In our study, obstructive processes may result from a decrease in airway wall integrity, or reduced lung elasticity. A developmental effect or direct toxic effect could then probably be the etiological factor.

Further research is needed to elucidate the findings presented in this manuscript.

## 5. Conclusions

While the decrease in lung function in relation to increasing prenatal and lactational dioxin exposure observed in childhood is no longer visible in adolescence, a decrease in lung function is now seen in relation to increasing BDE exposure in the teenagers. This is a novel finding and certainly warrants further research.

## Figures and Tables

**Table 1:** Descriptive statistics of the cohort.

*N* = 33 (18 Girls, 15 Boys)	Median	Mean	Range
Age (years)	14.3	15.0	14.0–18.7
Prenatal PCDD/F exposure I-TEQ (pg/g lipid)	29.8	32.6	9.05–88.8
Lactational PCDD/F exposure (I-TEQ (ng))	45.9	66.9	4.34–279
Serum PCDD/F (WHO 2005) TEQ (pg/g lipid)	1.6	2.2	0.4–6.1
Serum dl-PCBs (WHO 2005) TEQ (pg/g lipid)	1.8	2.2	0.04–7.8
Serum sum-BDEs (ng/g lipid)	9.9	14	4.9–73.6

PCDD: polychlorinated dibenzo-p-dioxins, PCDF: polychlorinated dibenzofurans, dl-PCBs: dioxin-like polychlorinated biphenyls (PCBs), BDEs: brominated flame retardants, TEQ: toxic equivalents.

**Table 2:** Association of serum totalTEQ (PCDD/F+dl-PCBs) (pg/g fat) with lung function parameters.

Spirometry		Diffusion Measurements		Body Box	
VC MAX	β: 0.49 *	TLCO SB	β: 0.11	VC MAX	β: 0.479 *
FEV 1	β: 0.63 *	VA	β: 0.392	TLC	β: 0.261
FEV1/VC MAX	β: 0.11	VIN	β: 0.459 *	RV	β: −0.2
PEF	β: 0.43			RV%TLC	β: −0.45
FEF 50	β: 0.36			FRC	β: −0.16

VC MAX: maximum vital capacity, FEV1: forced expiratory volume in 1 s, PEF: peak expiratory flow, FEF50: forced expiratory flow 50%, TLCO: transfer factor for carbon monoxide VA: alveolar volume, VIN: inspired volume, TLC: total lung capacity, RV: rest volume, FRC: functional residual capacity. *: p < 0.05. β: standardized regression coefficient.

**Table 3:** Correlation of serum sumBDE (ng/g fat) with lung function parameters.

Spirometry		Diffusion Measurements		Body Box	
VC MAX	(ρ): 0.27	TLCO SB	(ρ): 0.211	VC MAX	(ρ): 0.282
FVC	(ρ): 0.208	VA	(ρ): 0.212	TLC	(ρ): 0.084
FEV 1	(ρ): −0.539 *	VIN	(ρ): 0.243	RV	(ρ): −0.272
FEV1/VC MAX	(ρ): −0.575 *			RV%TLC	(ρ): −0.341
PEF	(ρ): −0.171			FRC	(ρ): −0.169
FEF 50	(ρ): −0.699 **				

VC MAX: maximum vital capacity, FVC: forced vital capacity, FEV1: forced expiratory volume in 1 s, PEF: peak expiratory flow, FEF50: forced expiratory flow 50%, TLCO: transfer factor for carbon monoxide VA: alveolar volume, VIN: inspired volume, TLC: total lung capacity, RV: rest volume, FRC: functional residual capacity. *: p < 0.05, ** p < 0.01. (ρ): Spearman’s correlation coefficient.
